# Association of Physical Exercise and Locus Coeruleus-Salience Network Functional Connectivity

**DOI:** 10.1093/geroni/igaf122.3712

**Published:** 2025-12-31

**Authors:** Nethra Rajeshkumar, Mali Madison, Madeline Stefano, Emily Jon, Sean Lawrence, Elayna Seago, Tae-Ho Lee, Benjamin Katz

**Affiliations:** Virginia Tech, Blacksburg, Virginia, United States; Virginia Tech, Blacksburg, Virginia, United States; Virginia Tech, Blacksburg, Virginia, United States; Virginia Tech, Blacksburg, Virginia, United States; Virginia Tech, Blacksburg, Virginia, United States; Virginia Tech, Blacksburg, Virginia, United States; Virginia Tech, Blacksburg, Virginia, United States; Virginia Tech, Blacksburg, Virginia, United States

## Abstract

Physical exercise is a modifiable lifestyle factor that supports brain health and cognition. The locus coeruleus (LC), the brain’s primary source of norepinephrine, plays a critical role in attention, and is among one of the first sites affected by Alzheimer’s-related pathology. Prior research suggests exercise promotes LC integrity, plasticity, and LC-dependent memory improvement. The salience network (SN), including the dorsal anterior cingulate cortex (dACC) and anterior insula, is crucial for identifying and responding to behaviorally relevant stimuli. LC-SN functional connectivity is thought to support efficient attentional control. Investigating the association between exercise and LC-SN connectivity may provide insight into neural resilience in the aging brain in a region implicated in Alzheimer’s pathology. Associations between LC-SN functional connectivity, response time, and exercise were compared in 43 older adults (65% female). fMRI data and response times were collected as participants completed the Place Discrimination Task (PDT), a measure linked to memory and attention. Weekly exercise was assessed via self-report. Linear regression models were used to examine the association between exercise and LC-SN connectivity and exercise and response time while controlling for age, sex, and family history of Alzheimer’s disease. The association between exercise and LC-dACC connectivity was marginally significant (β= .34, p = 0.0717). The association between exercise and LC-insula connectivity was not significant (β= -.03, p = 0.8481). However, task response time was significantly associated with exercise (β= -.50, p = 0.005). These findings suggest that while exercise may be linked to cognition during aging, associations with LC-SN functional connectivity are more tenuous.

